# The Role of GDF-15 in Heart Failure and Biomarker Potential—From Basic Science to Clinical Praxis

**DOI:** 10.3390/biology15060516

**Published:** 2026-03-23

**Authors:** Mário Barbosa, Maria Ana Martins, Joana Fernandes-Silva, Ana Melício, Álvaro M. Martins

**Affiliations:** 1 Department of Internal Medicine, Lusíadas Hospital Lisbon, 1500-458 Lisbon, Portugal; 2 Faculty of Medicine, University of Lisbon, 1649-028 Lisbon, Portugal; 3 Environmental Health Institute-ISAMB, TERRA Associated Laboratory, 1649-028 Lisbon, Portugal; 4 Department of Internal Medicine II, North Lisbon University Hospital Center—ULS Santa Maria, 1649-028 Lisbon, Portugal; 5 Institute for Research and Innovation in Health (i3S), University of Porto, 4200-135 Porto, Portugal; 6 Institute of Molecular Pathology and Immunology (IPATIMUP), University of Porto, 4200-135 Porto, Portugal

**Keywords:** GDF-15, heart failure, prognosis, diagnosis

## Abstract

Heart failure (HF) prognosis, particularly readmission and mortality, remains poor irrespective of advances in its management. Growth differentiation factor-15 (GDF-15) is a member of the transforming growth factor-beta (TGF-β) superfamily that arises as a promising biomarker to improve HF management, still despite two decades of extensive investigation some issues remain controversial. We performed a search in PubMed using the following Medical Subject Headings (MeSH) to identify relevant studies published in the last five years (after the latest updates of the guidelines by major Scientific Societies): “Growth differentiation factor-15 (GDF-15)”, “heart failure”, “prognosis” and “diagnosis”. The search was supplemented by data previous to this period, considered of utmost importance. A total of 115 articles written in English were retrieved. Extensive evidence supports GDF-15 as an independent predictor of adverse outcomes across the heterogeneous HF spectrum and its addition to risk scores based on traditional biomarkers improves prognostic power. However, limited tissue specificity restricts its diagnostic value. Tailored treatment strategies and its role as a therapeutic target remain speculative, as the effect of HF therapies on GDF-15 levels is unclear and clinical trials have been unsuccessful. Large prospective studies are needed to validate its reliability for routine clinical use.

## Simple Summary

Despite state-of-the-art management heart failure prognosis remains poor, resulting in a tremendous burden on health-care systems. Growth differentiation factor-15, a member of the transforming growth factor-beta superfamily, emerges as a promising biomarker to refine heart failure management. We performed a review focusing on its role in heart failure management based on contemporary evidence (i.e., after the latest updates of the guidelines by major Scientific Societies), and on previous articles (for purposes of historical contextualization or due to its indubitable importance). An extensive body of evidence supports this stress cytokine as an independent prognosticator of adverse outcomes throughout the wide spectrum of heart failure patients. Moreover, its incorporation into several risk scores based on traditional biomarkers has further improved their accuracy. Regarding diagnostic purposes, its lack of tissue specificity hinders its utility. Tailored treatment based on growth differentiation factor-15 variations during follow-up and its application as a therapeutic target are still hypothetical, as the effect that evidence-based heart failure drugs exert on growth differentiation factor-15 concentrations is poorly understood, and clinical trials targeting growth differentiation factor-15 have failed. Large-scale prospective studies are urged to confirm the trustworthiness of this biomarker, in order to enable its general routine clinical usage.

## 1. Introduction

**This section will introduce the concept of heart failure, its epidemiology, and the need for effective biomarkers for early detection, prognosis, and therapeutic interventions.**

Heart failure represents a complex clinical syndrome defined by the heart’s inability to maintain adequate blood flow necessary for the needs of the body, resulting from both structurally and functionally related abnormalities of ventricular filling and blood ejection [1,2]. Its presentation may manifest with fatigue, dyspnea, impaired exercise capacity, and edema [2]. Oftentimes, HF represents the culmination of other cardiovascular (CV) illnesses, including ischemic heart disease, hypertension, diabetes, and valvular heart disease [3,4,5,6]. The global burden of HF has grown to epidemic levels, with more than 64 million people being afflicted [7,8]. In the United States of America, HF accounts for nearly 1 million hospitalizations annually, with its related economic burden expected to surpass 70 billion dollars in 2030 [9]. Despite the advancements in pharmacological interventions and devices, the prognosis for HF patients remains dismal, with 50% of these patients expected to succumb to HF 5 years after being diagnosed [7,8].

The epidemiological context of HF presents some alarming trends. The occurrence of this disease rises with age, and its prevalence keeps climbing as the population ages. Nearly 10% of people 65 years and above suffer from HF, and the growth of the elderly cohort of people around the world supports this upward movement [8]. Also, the increase in associated comorbidities, including diabetes and obesity, contributes to the increasing burden of HF [5,8]. Given the huge morbidity, mortality, and economic burden linked with HF, there is a critical need for enhanced approaches for its earlier identification, risk stratification, and treatment [1].

One of the greatest challenges in the clinical management of HF is identifying patients with a higher risk of disease exacerbation and poorer outcomes in a timely and precise fashion. Today, in clinical practice, biomarkers, i.e., biomolecules that mirror certain pathophysiologic processes, are critical for diagnosing, prognosticating, and treating HF [2,10]. The natriuretic peptides, B-type natriuretic peptide (BNP), and N-terminal proBNP (NT-proBNP) are commonly used in clinical practice [2,10]. The peptides are produced from cardiac myocytes in response to increased wall stress and are critical for diagnosing HF and quantifying the probability of future events [11]. The efficacy and accuracy of such biomarkers, however, are limited and can be impacted negatively by age, renal function, and obesity, and therefore can be inaccurate in some populations of patients [2,11]. Thus, there is a critical need for novel biomarkers with the possibility of complementing the present gold standard for diagnosing HF and its severity [1,2].

Recent attention has shifted to growth differentiation factor-15 (GDF-15), identified as a member of the transforming growth factor-beta (TGF-β) superfamily, and considered a potentially useful marker for CV disease and HF [1,2,12]. Originally defined as a cytokine participating in the responses of tissues to stress, GDF-15 has been identified with a wide range of biological processes, such as oxidative stress, inflammation, apoptosis, and remodeling, all of them being key factors in the HF’s pathophysiology [1,5]. Evidence suggests that patients with chronic heart failure (CHF) and acute heart failure (AHF) present higher levels of GDF-15, and research has suggested that this marker could be of greater prognostic value compared with conventional surrogate indicators such as BNP and NT-proBNP [2,5].

Furthermore, GDF-15 levels correlate with worse clinical outcomes, including higher hospital admission rates and mortality rates; therefore, its use has been considered a future strategy for treatment and risk stratification [2,11,12].

We performed a search in PubMed using the following MeSH to identify relevant studies published in the last five years: “Growth differentiation factor-15 (GDF-15)”, “heart failure”, “prognosis” and “diagnosis”. Given that the European Society of Cardiology (ESC) published HF guidelines in 2021 and that the American College of Cardiology/American Heart Association (ACC/AHA) released new HF guidelines in 2022, and, subsequently, novel drugs and strategies were introduced for the management of this syndrome, we considered that an up-to-date review required scientific evidence that already incorporated those changes. Moreover, a manual screening of the bibliographic reference lists of relevant articles was implemented to identify additional data. The comprehensive literature retrieval was limited to articles written in English.

Additionally, we included some studies beyond this period of time for purposes of historical contextualization or considered as of indisputable relevance. A total of 115 articles were retrieved, from which we developed our review.

This review aims to explore the emerging role of GDF-15 as a biomarker in HF. The physiological action of GDF-15, its relationship with HF outcomes, and its future use for improving the identification, prognosis, and treatment of HF patients will be discussed. In addition, we will explore the possibility of GDF-15 helping with a greater understanding of HF mechanisms and causality and suggesting novel targets for future treatment.

## 2. Pathophysiology of Heart Failure

**This section will elucidate the pathophysiological mechanisms underlying heart failure, encompassing the complex interplay of hemodynamic changes, neurohormonal activation, myocardial remodeling and inflammatory processes contributing to its progression.**

The underlying pathophysiology of the HF syndrome involves a multifactorial process, including hemodynamic alterations, neurohormonal activation, myocardial remodeling and inflammatory responses, all of which contribute to the progression and worsening of the disease [13].

### 2.1. Hemodynamic Changes

The hallmark of HF is the impaired cardiac output, which can arise from either systolic or diastolic dysfunction. In systolic HF [i.e., heart failure with reduced ejection fraction (HFrEF)], there is a reduction in the heart’s ability to contract effectively, often due to ischemic heart disease, myocardial infarction or dilated cardiomyopathy. In contrast, diastolic HF [i.e., heart failure with preserved ejection fraction (HFpEF)] is primarily driven by impaired ventricular relaxation and increased stiffness, leading to inadequate filling during diastole [13].

Compensatory mechanisms are triggered in response to decreased cardiac output, primarily through the Frank–Starling mechanism, where increased ventricular preload attempts to maintain stroke volume. However, persistent elevated preload leads to ventricular dilation and increased wall stress, exacerbating cardiac dysfunction. Simultaneously, increased systemic vascular resistance (afterload) due to neurohormonal activation further impairs cardiac performance [13].

### 2.2. Neurohormonal Activation

Neurohormonal systems play a pivotal role in the progression of HF. The renin–angiotensin–aldosterone system (RAAS), sympathetic nervous system (SNS) and natriuretic peptides are key components in this response. Initially, activation of these systems is compensatory, aimed at maintaining perfusion to vital organs by increasing heart rate, contractility and vascular tone. However, chronic overactivation becomes detrimental [14].

- RAAS Activation: in response to decreased renal perfusion, the RAAS promotes vasoconstriction via angiotensin II, increases sodium and water retention through aldosterone, and induces fibrosis and hypertrophy in the myocardium, leading to worsening HF [14].
- SNS Activation: The SNS is activated to maintain cardiac output by increasing heart rate and contractility. However, prolonged stimulation leads to increased myocardial oxygen demand, arrhythmias and downregulation of β-adrenergic receptors, contributing to cardiac dysfunction [15].
- Natriuretic Peptides: Released in response to ventricular stretch, these hormones counteract the effects of the RAAS by promoting vasodilation, diuresis and natriuresis. However, in advanced HF, their compensatory effects are often overwhelmed [14].

### 2.3. Myocardial Remodeling

Myocardial remodeling is a central feature in the progression of HF, driven by neurohormonal and mechanical stress. This process involves changes in cardiac structure, cellular composition and function, ultimately leading to progressive ventricular dysfunction. Remodeling is characterized by:

- Cardiomyocyte hypertrophy: In response to increased workload, individual cardiomyocytes enlarge to maintain cardiac output. However, this hypertrophy is often maladaptive, leading to increased stiffness and diastolic dysfunction [16,17].
- Fibrosis: Activated by factors such as angiotensin II and aldosterone, myocardial fibrosis increases ventricular stiffness, impairs relaxation and contributes to diastolic dysfunction [16,17].
- Alterations in extracellular matrix: The degradation and disorganization of the extracellular matrix leads to weakened structural integrity, further exacerbating HF progression [16,17].

### 2.4. Inflammatory Processes

Inflammation plays a crucial role in HF, especially in HFrEF, where pro-inflammatory cytokines such as tumor necrosis factor-alpha (TNF-α), interleukin-1 (IL-1), and interleukin-6 (IL-6) are upregulated. These cytokines contribute to myocyte apoptosis, fibrosis and endothelial dysfunction, all of which accelerate myocardial damage. Moreover, chronic inflammation has been associated with impaired nitric oxide bioavailability, contributing to endothelial dysfunction and promoting vasoconstriction [18].

Growth differentiation factor-15 has emerged as a significant biomarker and contributor to the pathophysiology of HF. Elevated levels of GDF-15 are associated with adverse outcomes in patients with HF, particularly in the context of myocardial ischemia and remodeling [19]. This cytokine is released in response to stress signals and inflammatory processes, acting as a protective factor against cellular injury while also indicating myocardial stress and dysfunction [19].

The continuous elevation of GDF-15 not only reflects the ongoing pathological processes but also serves as a prognostic indicator of disease progression and mortality risk in HF patients [20].

## 3. GDF-15: Overview and Molecular Mechanisms

**This segment will provide an in-depth exploration of GDF-15, highlighting its physiological role, cellular sources, signaling pathways and the mechanisms through which it exerts its effects on cardiac function and remodeling.**

Growth differentiation factor-15 belongs to the TGF-β superfamily and has been identified earlier as a macrophage inhibitory cytokine (MIC-1) [21,22]. However, its wide-ranging functional roles were identified in different physiological settings and disease states, including CV disease [21,23]. The low baseline levels under normal physiological settings, compared with the higher levels under cellular stress, inflammation, ischemia, and cardiac overload, highlight its relevance as a marker for many disease processes [23]. Particularly, in the healthy myocardium, GDF-15 expression is generally low, whereas in HF it is markedly upregulated in response to cellular stress in several cardiac cell populations. These include, cardiomyocytes, endothelial cells, macrophages, and fibroblasts, reflecting myocardial injury and inflammatory signaling rather than a single compartment-specific source [24,25,26].

Growth differentiation factor-15 may influence multiple cardiac and vascular cell types in HF, including cardiomyocytes, endothelial cells, vascular smooth muscle cells, fibroblasts and immune cells, where it modulates stress-response pathways, inflammatory signaling and extracellular matrix remodeling [26,27]. Through these mechanisms, GDF-15 may contribute to intercellular crosstalk between myocardial, vascular and immune compartments that collectively regulate cardiac remodeling and disease progression.

### 3.1. Cellular Sources and Regulation

Growth differentiation factor-15 is widely present in the heart, liver, kidney, and adipose tissues, and its major sources are cardiomyocytes, endothelial cells, and macrophages [24]. Growth differentiation factor-15 is regulated in response to a range of stimuli such as endoplasmic reticulum stress, oxidative stress, hypoxia, and pro-inflammatory cytokines TNF-α and IL-6 [23]. Also, mitochondrial dysfunction, mainly via the pathway of the integrated stress response (ISR), has been linked with GDF-15 levels, hence forming a link between its presence and cellular metabolic stress, and the general energy homeostasis of the organism [23].

### 3.2. Signaling Pathways and Mechanistic Insights

Unlike other superfamily members of TGF-β, whose action mainly occurs via traditional suppressor of mothers against decapentaplegic homolog (Smad)-mediated pathways, GDF-15 has been more and more widely noted to act via the glial cell line-derived neurotrophic factor (GDNF) receptor family member GDNF family receptor alpha-like (GFRAL), whose presence has been notably identified in the brainstem [22]. The interaction of GDF-15 and GFRAL signaling profoundly regulates CV and metabolic processes via complex neural networks affecting appetite and restoration of energy balance [23].

Recent studies have indicated that GDF-15 can promote cardiac remodeling, independently of Smads, directly engaging phosphoinositide 3-kinase/protein kinase B (PI3K/Akt), extracellular signal-regulated kinases 1/2 (ERK1/2), and nuclear factor kappa-light-chain-enhancer of activated b cells (NF-κB) signaling cascades, necessary for survival, proliferation, and fibrosis in response to injury [21,23]. Elevated levels of GDF-15, measured in HF models, correlate with beneficial cardiac outcomes, reducing excessive inflammatory responses and limiting harmful remodeling, albeit context-dependent [24]. Growth differentiation factor-15 also regulates extracellular matrix remodeling via modulation of matrix metalloproteinases (MMP) and tissue inhibitors of metalloproteinases (TIM), critical to its roles in adaptive and maladaptive cardiac responses [21,23]. 

Circulating non-coding RNAs may also interact with GDF-15–related pathways in HF. Several studies have reported significant inverse correlations between GDF-15 levels and miR-106a-5p and miR-223-3p [28,29]. Moreover, epigenetic regulation of the miR-21 promoter and direct binding of miR-21 to GDF15 mRNA further support the existence of regulatory RNA networks influencing GDF-15 expression and associated signaling pathways [30].

### 3.3. Effects on Cardiac Function, Heart Failure and Remodeling

Growth differentiation factor-15 has been proven to induce both beneficial and harmful impacts on cardiac tissue. Particularly, its anti-inflammatory and anti-fibrotic properties are critical in protecting against cardiac injury in periods of acute stress, such as myocardial infarction, through prevention of leukocyte invasion and maintaining the function of contraction [23]. In addition, its antioxidant action, including inhibition of superoxide dismutase (SOD) and catalase activity, contributes significantly towards developing the heart’s resistance against ischemic/reperfusion injury [21]. However, chronic overexpression of GDF-15 has been associated with negative cardiac remodeling processes, including fibrosis and left ventricular hypertrophy, possibly due to its pro-apoptotic action on myocytes and its action on extracellular remodeling of the extracellular matrix [23].

In the context of HF, GDF-15 has been the target of significant research into its dual function of being both a disease modifier and a marker. Elevated levels of circulating GDF-15 correlate with disease severity, left ventricular dysfunction, and poor clinical outcomes, hence classifying it as a prognostic marker [24]. Mechanistically, GDF-15 has been identified for its capacity to modulate myocardial response under stressed conditions, therefore protecting against excessive inflammation and maladaptive cardiac hypertrophy, especially in the earlier phases of HF. In contrast, in established phases of HF, chronic elevation of GDF-15 levels has been associated with the occurrence of cachexia, the disruption of systemic metabolism, and cardiac dysfunction, highlighting the complex and context-specific roles of GDF-15 [23].

Growth differentiation factor-15 has been recognized as a stress-responsive protein induced by oxidative and mitochondrial stress [27]. Several studies suggest that it may exert cardioprotective effects by attenuating reactive oxygen species accumulation, preserving mitochondrial function, and limiting maladaptive mitophagy in injured cardiomyocytes [31,32]. Additionally, GDF-15 is associated with cardiometabolic stress and adipose tissue. Adipocytes and adipose-resident macrophages can contribute to circulating GDF-15 under metabolic stress, and epicardial adipose tissue has emerged as an important mediator of myocardial dysfunction and HF risk, particularly in obesity and type 2 diabetes [33,34,35]. Thus, increased GDF-15 may reflect not only myocardial injury but also extracardiac metabolic-inflammatory burden in HF. Nevertheless, the exact mechanism between adipose-derived GDF-15 and mitochondrial dysfunction in HF remains to be fully elucidated. Moreover, GDF-15 contributes to endothelial dysfunction and vascular remodeling, both of which being main factors of HF pathophysiology. Growth differentiation factor-15 impacts vascular homeostasis and myocardial perfusion, connecting its action with the progression of HF, via modulation of endothelial nitric oxide synthase (eNOS) activity and the production of pro-inflammatory cytokines [21,23]. The two-way relationship between HF and metabolic insults, including insulin resistance and mitochondrial dysfunction, sets GDF-15 on a key intersection of CV and metabolic disease, and therefore, places it as a promising target for treatment [24].

## 4. GDF-15 as a Biomarker in Heart Failure

**This section will focus on the clinical utility of GDF-15 as a biomarker in heart failure. It will review the evidence supporting its association with disease severity, diagnosis prognosis and risk stratification.**

### 4.1. GDF-15 a Forever Emerging Biomarker

Growth differentiation factor-15 was described for the first time as a promising biomarker of CV disease two decades ago by Kempf and colleagues [36].

Following in vivo studies in CHF patients identified a proportional risk between the concentration of GDF-15 and all-cause mortality. Relevantly, a relationship between GDF-15 measurements ischemic etiology, higher New York Heart Association (NYHA) functional class, impaired left ventricular ejection fraction (LVEF) and N-terminal pro-B-type natriuretic peptide (NT-proBNP) levels was also reported [37].

Despite being a marker of systemic inflammation, as GDF-15’s synthesis is triggered by unspecific inflammatory stimuli, its circulating concentration is, notoriously, augmented in HF [38] as an adaptive response aiming to minimize myocardial stress and cardiac remodeling [36,39,40,41].

Additionally, GDF-15 is expressed in hypertrophic and dilated cardiomyopathy in response to volume overload, ischemia and HF [42]. Therefore, the rationale for the application of this pleiotropic protein in HF is based on its release due to cardiac insult [43,44,45,46,47].

Modern investigations have proposed that left ventricle (LV) dimensions and LVEF are associated with GDF-15, which is consistent with its role in cardiac remodeling through fibrosis and inflammation [47]. Moreover, some studies have emphasized the anti-inflammatory properties of GDF-15 in multiple organs (e.g., heart, kidney, liver and lungs) [48,49].

Apart from the aforementioned functions, GDF-15 also intervenes in bone regulation since it abrogates osteocyte differentiation in response to hypoxia, and in cancer and spondylarthritis [50,51].

The protein-receptor mechanisms through which GDF-15 mediates its biological effects have yet to be fully explained [49,52].

In the context of HF, due to its multifaceted function, the biological pathways underlying elevated GDF-15 concentrations remain poorly defined [53].

Emmerson et al. described for the first time the endogenous receptor for GDF-15, the GFRAL receptor [54]. This relevant finding may contribute to elucidate the pluriorganic role of GDF-15 and facilitate future applications in different conditions [55].

The fact that the only recognized receptor for GDF-15, GFRAL, is exclusively located in the brain, added to its role in a wide range of pathways in distinct tissues, generates a reasonable doubt regarding its specific involvement in cardiac intracellular and intercellular mechanisms [26].

Unfortunately, preclinical and clinical investigations have provided controversial data regarding the role of GDF-15 in cardiac pathophysiology. It remains uncertain whether GDF-15 performs a cardioprotective effect against cell death, ischemic scar development and hypertrophy, or exhibits a deleterious impact through fibrosis, ischemia and atrophy [55].

To clarify this matter, the circulating concentration of 363 biomarkers was analyzed in 2279 patients selected from the BIOlogy Study to TAilored Treatment in Chronic Heart Failure (BIOSTAT-CHF) trial [53]. The authors concluded that in the setting of HF and high plasma values of GDF-15, the activation of inflammatory pathways and pathways associated with insulin-like growth factor (IGF-1) receptor signaling and tissue, bones and branching structures, remodeling was typical [53]. Pathway over-representation analysis demonstrated that the main up-regulated biomarkers were fibroblast growth factor 23 (FGF-23), death receptor 5 (TRAIL-R2), WNT1-inducible signaling pathway protein 1 (WISP-1), tumor necrosis factor receptor superfamily member 11a (TNFRSF11A), leucocyte immunoglobulin-like receptor subfamily B member 4 (LILRB4) and trefoil factor 3 (TFF3). Ceelen D et al. recognized that high concentrations of GDF-15 implicated an increased risk of two-year all-cause mortality and HF hospitalization. Furthermore, upper GDF-15 quartiles correlated with lower weight [53], which may be justified by the fact that GDF-15 triggers anorexia by binding to GDNF/GFRAL in the nucleus of the solitary tract and the area postrema, inhibiting appetite [56].

Given that GDF-15 represents wall stiffness due to inflammatory injury, it may yield complementary pathophysiological information to that of natriuretic peptides’ (which signify hemodynamical wall distress) and troponins’ (which represent myocardial cell lesions) [57].

Heart failure management based on biomarker quantification stands on the premise that variations over time reproduce clinical improvement or disease progression, as biomarkers represent a product of a specific pathophysiological axis. Nonetheless, the application of analytical information in clinical practice is complex [10,53,58].

A myriad of biomarkers have been extensively investigated for diagnostic, prognostic and, even, therapeutic target purposes. Yet, emerging biomarkers still play a supplementary role in traditional HF management, as natriuretic peptides and troponins remain the gold standard biomarkers in HF [1,59,60]. Nevertheless, the current evidence seems to favor some promising candidate biomarkers concerning prognosis.

Biological variability (i.e., the expected variability within an individual during disease progression) plays a pivotal role in a biomarkers’ utilization, as it represents the reliability to consistently reproduce the results [58,61,62].

In the particular case of GDF-15, previous authors mention that it has a low biological variability [44,63,64,65] and recent studies point GDF-15 as a more trustworthy biomarker than the classical biomarkers NT-proBNP, high-sensitivity cardiac troponin T (hs-cTnT), and high-sensitivity C-reactive protein (hs-CRP) [66].

Nevertheless, the European Society of Cardiology (ESC) Biomarker Study Group of the Heart Failure Association (HFA) states that GDF-15 varies significantly overtime compared to soluble isoform of suppression of tumorigenicity 2 (sST2) and galectin-3 (Gal-3) and therefore its variability along distinct stages in disease evolution hinders its use [60].

Growth differentiation factor-15 is considered a biomarker of senescence as its levels increase with aging in healthy individuals [44,67]. Adding to this matter, age is a universal non-modifiable CV disease risk factor; therefore, it may appear problematic to distinguish if the upregulation of GDF-15 and its prognostic meaning are a product of natural senescence or disease severity [47]. To address this issue, an explorative post hoc study of the Supplemental Benefit of an ARB in Hypertensive Patients with Stable Heart Failure Using Olmesartan (SUPPORT) trial examined GDF-15 in 942 Japanese hypertensive patients with compensated CHF (93% in NYHA class II) with a median age of 68 years, ranging from 50 to 79 years [47]. Despite median GDF-15 values being increased along age groups, the prognostic accuracy of the composite endpoint of HF hospitalization or all-cause mortality was not affected in a long-term mean follow-up of 8.6 years [47].

Another Japanese investigation revealed that, besides correlating with age, GDF-15 augmented with progressing NYHA functional class in a CHF cohort encompassing different HF phenotypes [68].

Apart from age, multiple factors increase circulating GDF-15, namely, male gender, infection, and renal dysfunction; on the other hand, BMI seems to vary inversely with GDF-15 in HF cohorts [60].

Welsh and collaborators measured GDF-15 in 19,462 individuals from the Generation Scotland Scottish Family Health Study (GS:SFHS) and verified a similar median value between sexes, a slow rise up to 50 years of age, and a greater increase above the age of 50 [67]. In this general population cohort, GDF-15 was associated with older age, smoking, cancer, heart disease, HF, stroke, diabetes and renal impairment. Concerning other biomarkers, a positive relation with NT-proBNP and high sensitivity troponin was acknowledged. Contrarily to Meijers et al. [60], GDF-15 circulating serum levels showed a positive correlation with BMI. The highest GDF-15 levels were observed in pregnant women [67], justifying GDF-15’s alternative designations as placental transforming growth factor-beta (pTGFB) and placental bone morphogenetic protein (PLAB). Interestingly, a correlation with socioeconomic deprivation was also described [67].

Concerning renal function, a post hoc analysis of the Prospective comparison of ARNI with ACEI to Determine Impact on Global Mortality and morbidity in Heart Failure (PARADIGM-HF) trial demonstrated a correlation between baseline GDF-15 and creatinine in HFrEF patients [64] and a cohort of HFpEF patients found that GDF-15 was a solid predictor of renal dysfunction progression [69]. Lewis and colleagues recognized that higher baseline GDF-15 correlated with male sex, higher NT-proBNP, diabetes, and renal dysfunction in an HFpEF population [69].

A 2025 Brazilian study found that GDF-15 is better correlated with indicators of renal dysfunction than NT-proBNP in CHF patients. The investigators propose GDF-15 as an early predictor of kidney disease progression based on the assumption that impaired tissue perfusion leads to neurohormonal activation by the kidneys, which triggers GDF-15 production prior to obvious congestion and natriuretic peptide synthesis [70].

### 4.2. Diagnosis

Kempf et al. originally considered that due to the fact that GDF-15 is non-cardiac-specific, it would not be adequate for diagnostic purposes [37].

Given that GDF-15 is a stress induced cytokine expressed in several conditions, it is questionable if its upregulation represents a severe clinical scenario rather than just HF [71].

A systematic review concluded that the fibrosis biomarkers GDF-15, Gal-3 and sST2 can distinguish patients with HFpEF from individuals without HF but cannot discriminate HFpEF from HFrEF [72].

A study performed in Eastern Europe concluded that AHF patients admitted to an emergency service had higher GDF-15 levels compared to ambulatory compensated CHF patients. Additionally, GDF-15 was as accurate as NT-proBNP and high-sensitive troponin for diagnosing AHF and superior for prognosticating short-term fatalities (in-hospital and 30-day mortality) [73].

A 2023 meta-analysis of 1550 patients, from a total of ten studies consulted in both Chinese and English literature, proposed GDF-15 as a novel biomarker for diagnosing HFpEF due to its high sensitivity and specificity [74].

A current systematic review and meta-analysis from 2025, involving a total of 5696 HFpEF patients in both AHF and CHF settings, acknowledged that the diagnostic accuracy of HFpEF by GDF-15 was superior to that of NT-proBNP. The authors underscored that GDF-15 was effective in diagnosing HFpEF in the subgroup of obese patients [75]. Interestingly, the combination of biomarkers did not improve the capability from distinguishing HFpEF patients from controls [75].

Heart failure with preserved ejection fraction accounts for around half of the global HF population, evolving with 5-year mortality and HF rehospitalization rates similar to HFrEF [76]. The diagnosis of this phenotype is sometimes problematic with natriuretic peptides, especially in the obese (who express lower values) [76], which may enable an opportunity for emerging biomarkers. Atrial fibrillation and renal failure, frequent comorbidities in HFpEF, may also be confounders as the stretch markers are heightened to an extent that may not relate exclusively to HF in such conditions [75].

However the Biomarker Study Group of the HFA maintains that GDF-15 is unfit for diagnostic purposes since it is dubious if its upregulation means HF worsening/progression or another disease or even general deterioration [60].

### 4.3. Prognosis

The main drive for GDF-15 investigation in clinical trials has been the prognostication of CV outcomes in coronary arteriopathy disease [66].

Despite hypothetical cardioprotective properties postulated in initial basic studies, namely when GDF-15 was described by Bootcov and collaborators in 1997 for the first time [40], increased GDF-15 circulating concentrations have been consistently associated with negative outcomes in patients with CV disease, as well as in seemingly healthy individuals from population-based samples [77].

The superior limit of GDF-15 in healthy individuals is 1200 pg/mL [45].

There is a substantial debate apropos of the GDF-15’s threshold in CV disease risk stratification. A recent individual patient meta-analysis of eight studies, including more than 50,000 patients with atherosclerotic CV disease, recognized that GDF-15 was an independent predictor of CV death and HF hospitalization. Also, the risk increased along with GDF-15 concentration terciles (<1200 pg/mL, 1200–1800 pg/mL, >1800 pg/mL) [78].

A CHF investigation established an optimal cut-off for predicting composite outcome of HF readmission and global mortality of 1400 pg/mL [47].

Another 2024 study performed receiver operating characteristic (ROC) analysis, finding an optimal GDF-15 cut-off value on admission of 5115.5 pg/mL to predict the composite endpoint of 1-year all-cause mortality or rehospitalization due to HF decompensation. The optimal cut-off at discharge and at the 30-day visit was 4145 pg/mL and 4218.5 pg/mL, respectively [79].

#### 4.3.1. Prognosis: Chronic Heart Failure Evidence

An analysis of the Valsartan Heart Failure Trial (ValHeFT) database, from 2010, was one of the pioneer investigations of the role of GDF-15 in HF prognosis. Circulating GDF-15 was measured at baseline and at 12 months in CHF patients during a median follow-up of 23 months. The promising results revealed that GDF-15 was independently associated with increased risk of all-cause mortality, although its circulating concentration did not decrease with valsartan [44].

Still, the interpretation of such conclusions required some prudence since there were concerns that the use of evidence-based treatment was low [80].

The biomarker substudy of the Heart Failure: A Controlled Trial Investigating Outcomes of Exercise Training (HF-ACTION) trial was a hallmark regarding the assertion of GDF-15 as a valid prognosticator of adverse events. This multicenter cohort of 910 CHF ambulatory patients with HFrEF was followed during a median of 30 months. This subanalysis demonstrated that GDF-15 was an independent predictor of all cause death [80].

Another relevant and more recent study performed by the investigators of the PARADIGM-HF, recognized that GDF-15 values did not decrease with sacubitril/valsartan in ambulatory CHF patients with HFrEF and that GDF-15 was an independent predictor of mortality and CV outcomes, namely HF hospitalization [64]. High GDF-15 was associated with advanced age, NYHA class III/IV, diabetes, abnormal renal function, NT-proBNP and hsTnT [64]. Bouabdallaoui and collaborators speculated that GDF-15 could represent a different physiopathological pathway since it was not influenced by sacubitril/valsartan and its prognostic power persisted significantly, even after considering other variables. Its additional and distinctive information seemed to relate to disease burden (i.e., surrounding comorbidities) rather than to HF itself [64]. This study showed that GDF-15 helps to identify at-risk patients on state-of-the-art treatment, highlighting its utility [64]. Subsequently, a total of 1559 CHF patients with HFrEF were selected from the PARADIGM-HF trial; GDF-15 predicted the primary composite endpoint, CV death, and all-cause mortality during a mean follow-up of 30.7 months [81].

The EMPagliflozin outcomE tRial in Patients With chrOnic heaRt Failure (EMPEROR) program, conducted in North America, Europe and Asia, found that patients with higher GDF-15 levels are usually older men with a longer duration of HF and with more severe symptoms, higher NT-proBNP levels, and renal impairment [82]. This post hoc exploratory analysis enrolled CHF of different etiologies with HFrEF or HFpEF, from the EMPEROR-Reduced and EMPEROR-Preserved trials, respectively, and verified that GDF-15 was an independent risk marker for HF hospitalizations during a 52-week follow-up [82].

Apart from large-scale studies, several contemporary meta-analyses and systemic-reviews have demonstrated the importance of GDF-15 in HF prognostication in a vast set of patients. As an example, a meta-analysis of ten studies, encompassing 6244 stable CHF patients, revealed that increased circulating GDF-15 concentrations prognosticated all-cause mortality in CHF of ischemic etiology, but not in those with a non-ischemic cause. In addition, the correlation was stronger after eliminating HFrEF individuals, suggesting that GDF-15 could have a better prognostic accuracy in HFpEF [83]. Furthermore, subgroup analysis demonstrated that the predictive power of GDF-15 did not vary with age, suggesting that GDF-15 could be an independent risk biomarker in CHF patients with different ages. Regarding NYHA functional class, seemingly, it did not interfere with GDF-15’s prognostic precision as no variability was recognized [83].

A 2024 systematic literature review by Javaheri et al. embracing a broad spectrum of HF patients and phenotypes concluded that circulating GDF-15 was an independent predictor of mortality risk (both all-cause and CV-related), hospitalization/rehospitalization, and other adverse outcomes including renal dysfunction, lower BMI and impaired exercise capacity [71].

Another systematic review and meta-analysis from 2025, involving a total of 5696 HFpEF patients in both AHF and CHF settings recognized that GDF-15 was able to predict the risk of all-cause mortality and HF hospitalization in this HF phenotype [75].

#### 4.3.2. Prognosis: Acute Heart Failure Evidence

A decade ago a post hoc analysis of the Serelaxin, recombinant human relaxin-2, for treatment of acute heart failure (RELAX-AHF) study was a breakthrough regarding the importance of GDF-15 in AHF prognosis since it had been evaluated basically in CHF [84]. The values of GDF-15 were quantified in 1161 AHF patients at different time-points (baseline and at days 2, 5, 14, and 60) [84]. Increases in GDF-15 values, but not baseline determinations, were associated with the composite endpoint of HF or renal failure readmission at 60 days/CV death or CV death at 180 days [84]. Additionally, GDF-15 correlated with male sex, worse LVEF, lower BMI, worse congestive symptoms, comorbidities (diabetes mellitus, ischemic heart disease, renal dysfunction, anemia, atrial fibrillation), hs-cTnT and NT-proBNP [84].

Given that it was a post hoc analysis the results were interpreted as hypothesis-generating [84].

A subsequent retrospective subanalysis of the RELAX-AHF trial reported that the evaluation of a multi-marker panel (including GDF-15) at the above-mentioned time points compared with a single-marker, single-time-point approach improved risk prediction of CV death and HF readmission in patients with AHF and mild-to-moderate renal dysfunction. The investigators hypothesized that a multi-marker strategy could better reproduce the syndrome’s progression and therefore enhance risk stratification and prevention of potential adverse outcomes through earlier intervention [85].

The utilization of a multiple-biomarker strategy may enhance risk stratification, diagnostic precision and heart disease management [86].

It seems that the assessment of multiple biomarkers at various time points improves death and rehospitalization risk stratification, according to a 2023 serially measured multi-marker analysis of the Translational Initiative on Unique and Novel Strategies for Management of Patients With Heart Failure (TRIUMPH) study, including 475 AHF patients, that disclosed an association between GDF-15 and all-cause mortality and HF hospitalization. A multi-marker model joining GDF-15, NT-proBNP, and troponin I improved risk stratification [87].

A small sample size prospective single-center investigation comprising 84 Thai patients admitted for AHF, predominantly HFpEF, found a correlation between GDF-15, collected upon admission and discharge, and HF readmission at 30, 90, and 180 days, and end of follow-up (median follow-up time of 213 days) [88]. Additionally, a relation between reduction in GDF-15 concentrations, from admission to discharge, and rehospitalization across all studied time points was also observed, emphasizing GDF-15’s properties as a dynamic biomarker that could reliably translate the course of AHF [88].

A single-center cohort of 104 patients admitted after an episode of HF decompensation also tested the performance of combined serial measurements of GDF-15 and NT-proBNP for risk stratification of 1-year all-cause mortality or HF rehospitalization [79]. Biomarkers were measured on admission, at discharge and after 30-day follow-up visit; during follow-up the concentrations of both biomarkers presented a downward kinetics [79]. The investigators concluded that combined serial measurement was superior to a single measurement in risk stratification, since it presented the best predictive value for the primary composite endpoint [79]. Notoriously, the 30-day follow-up time point was more precise for risk prediction, and small changes in the levels of GDF-15 and NT-pro BNP represented high-risk patients that did not respond to the treatment [79].

Post-stabilization concentrations reflect baseline GDF-15 and the actual disease status, and may, therefore, be more precise for risk stratification. The fact that a persistently high value of biomarkers, despite adequate treatment, has been related to impaired outcome [85] may warrant the assessment of biomarkers in the post-baseline period [79].

Yin et al. acknowledged that GDF-15 seems to be superior to NT-proBNP, as higher serum GDF-15, collected within 48 h of admission, predicted the risk of one-year mortality and HF readmission better than the classic biomarker in a cohort of HFpEF patients admitted due to AHF from the China Patient-centered Evaluative Assessment of Cardiac Events Prospective Heart Failure Study (China PEACE 5p-HF Study) [89].

A prospective, single-center study of 104 HFrEF patients hospitalized due to AHF, with a median follow-up of 23.5 months, concluded that GDF-15 and sST2 showed the highest predictive power for all-cause mortality, when compared to the clinical and biochemical variables, namely natriuretic peptides [90]. Table 1 summarizes the trials that addressed the prognostic utility of GDF-15.

#### 4.3.3. Incremental Value of Traditional Prognostic Scores

A novel biomarker should add information to the current ones [10].

The inclusion of GDF-15 in risk stratification models, overall, has refined outcome prediction.

In 1559 CHF patients with HFrEF selected from the PARADIGM-HF trial, GDF-15 amongst other biomarkers, was tested to see if it, individually or jointly, improved the precision of the PRognostic Evaluation During Invasive CaTheterization for Heart Failure (PREDICT-HF) model; neither of the biomarkers assessed individually or collectively enhanced the prognostic power for the primary composite endpoint, CV death and all-cause mortality [81]. Despite the fact that GDF-15 was found to be an independent predictor of the primary composite endpoint, CV death, and all-cause mortality during a mean follow-up of 30.7 months when evaluated individually, its addition to the prognostic model did not improve risk prediction provided by clinical and routine laboratory (namely natriuretic peptides) [81].

A study conducted in a department of Cardiology of a Chinese general hospital enrolling 823 patients suffering from CHF due to coronary heart disease, divided in three groups (27.9%) with HFrEF, (32.9%) with heart failure with mildly reduced ejection fraction (HFmrEF), and 39.1% with HFpEF, found a link between increased GDF-15 determinations and risk of all-cause mortality and HF readmission across all HF phenotypes during a 9.4 year follow-up [91]. Importantly, the integration of GDF-15 to the Meta-Analysis Global Group in Chronic Heart Failure (MAGGIC) risk score-based model (calculated from 13 variables) enhanced the prognostic precision of HF rehospitalization in the HFpEF subgroup, the group with the highest GDF-15 values [91].

The CardShock Investigators tested GDF-15 in severe AHF, i.e., cardiogenic shock, recognizing a link with 90-day mortality and improvement of the CardShock risk score with its incorporation [92].

An exploratory analysis of the ongoing Prospective mUlticenteR obServational stUdy of patIenTs with Heart Failure with preserved Ejection Fraction (PURSUIT-HFpEF) registry, reported that GDF15 correlated with the composite of all-cause mortality and HF hospitalization in an elderly (median age was 81 years) multimorbid cohort with HFpEF admitted for acute decompensated HF [93]. This cytokine was also strongly associated with inflammation, cardiac burden, anemia, renal dysfunction and malnutrition. Importantly, the incorporation of GDF-15 added incremental prognostic value to the MAGGIC risk score, reinforcing its utility as a complementary biomarker of traditional HF risk stratification scores [93].

The Heartmarker score is a novel risk stratification model that analyzed the combination of NT-proBNP, sST2 and GDF-15 from a cohort of 245 outpatients (encompassing HFrEF, HFmrEF and HFpEF) enrolled from the Heart Failure Classification (HaFaC) trial performed in the Netherlands [94].

The combination of these biomarkers was more accurate than the NYHA functional class, a routine index of HF severity preconized by the ESC [59] and the ACC/AHA [1] for the prediction of the composite endpoint event-free survival (HF hospitalization, appropriate implantable cardioverter-defibrillator shock or death) and was comparable for functional capacity estimation, evaluated by the 6 min walking test, within the 1-year follow-up [94].

It is expectable that soon GDF-15 will be incorporated in well-established HF prognostic multi-biomarker models; similarly to other conditions, namely the ABC (age, biomarkers, clinical history) bleeding score in atrial fibrillation patients undergoing anticoagulation [95,96].

#### 4.3.4. Comorbidities and Different Clinical Scenarios

Despite the absence of specificity and its fluctuation with numerous physiological factors (such as aging and pregnancy) and pathological conditions (including inflammation, atrial fibrillation, diabetes, anemia, renal dysfunction, and body weight), several large-scale clinical trials support that GDF-15 is a solid independent predictor of adverse heart failure outcomes across diverse clinical backgrounds.

In the previous literature addressing CHF patients, GDF-15 has been related to age, burden of comorbidities (renal dysfunction, diabetes), NYHA class/disease severity, and NT-proBNP levels [37,44].

A biomarker substudy of the hallmark anticoagulation trial Apixaban for Reduction in Stroke and Other Thromboembolic Events in Atrial Fibrillation (ARISTOTLE) acknowledged that in patients suffering from atrial fibrillation on oral anticoagulation higher levels of GDF-15 implicated an increased risk of death and of developing HF or worsening of pre-existing HF. Furthermore, GDF-15 values were highest in the HFrEF group and, also, the HF hospitalization and all-cause mortality rate [97].

A nested case–control analyses of the COmpare Rivaroxaban in Heart Failure: a Major Evaluation of Risk (COMMANDER) HF trial investigated whether proteomic biomarkers related to inflammation and remodeling predicted CV events in HFrEF patients with underlying coronary artery disease after a HF decompensation episode. Notably, GDF-15 was a strong predictor of the composite of CV events formed by HF hospitalization, stroke/myocardial infarction and sudden cardiac death. Despite being a reliable predictor of overall adverse CV events, GDF-15 was not event-specific, i.e., did not differentiate the type of adverse outcome [98].

The pivotal influence of GDF-15 in the cardiorrenal interface was supported by a post hoc analysis from the Canagliflozin Cardiovascular Assessment Study (CANVAS) trial as each doubling in baseline circulating GDF-15 was related to a higher risk of CV (including HF) and renal outcomes in a 4330 cohort of type 2 diabetes patients at high CV risk during a median follow-up of 6.1 years [99].

Given its central importance in multiple pathways, GDF-15 is considered a reliable biomarker in the cardiovascular-kidney-metabolic syndrome, a constellation of pathologies formed by CV disease, chronic kidney disease, obesity and diabetes [100].

Anemia is another frequent comorbidity in HF patients, the Reduction of Events by Darbepoetin alfa in Heart Failure (RED-HF) trial observed a link between baseline and change in GDF-15 during 6 months follow-up and the primary composite endpoint of all-cause mortality or HF hospitalization in CHF patients with HFrEF and anemia (hemoglobin level of 9.0–12.0 g per deciliter). Also, GDF-15 was inversely correlated with various indices of anemia and linked positively with ferritin [101], a renowned inflammatory surrogate marker [1,59].

Considering that dilated cardiomyopathy results from myocardial remodeling, which is inherently related to fibrosis and extracellular matrix expansion, Kayvanpour and colleagues explored if circulating GDF-15 could be an alternative to endomyocardial biopsy and cardiac magnetic resonance imaging for assessing myocardial fibrosis [102]. This investigation revealed that GDF-15 correlated with systemic inflammation, myocardial remodeling and overall disease burden. In fact, GDF-15 proved to enhance risk stratification through HF adverse events (i.e., death, hospitalization, sudden cardiac death, aborted sudden cardiac death, heart transplantation), and all-cause death prognostication in a median follow-up duration of 2.7 years [102]. Regarding patients’ characteristics, elevated GDF-15 values were significantly linked to older age, diabetes, higher BMI, renal dysfunction and impaired functional capacity. As for HF features, GDF-15 correlated proportionally to NT-proBNP levels and contrariwise with LVEF [102].

## 5. GDF-15 in Clinical Practice: Challenges and Opportunities

**Herein, the article will discuss GDF-15’s potential role in guiding therapeutic strategies and assessing treatment response. Additionally, it will explore the ongoing research and potential opportunities for enhancing its clinical applicability as a treatment target and future perspectives.**

The biological variation in a biomarker is critical for the interpretation of serial measurements; based on this premise Meijers and collaborators analyzed the variation in several biomarkers within a subject over time in CHF patients. The authors concluded that GDF-15 and NT-proBNP presented the highest biological variability compared to other biomarkers (e.g., Gal-3, sST2) and therefore less useful for patient follow-up and biomarker-tailored management [58].

Former studies verified that left ventricular assist device implantation decreased circulating GDF-15 in patients with advanced HF [103,104].

The Safety, Tolerability, and Efficacy of Rapid Optimization, Helped by NT-proBNP Testing, of Heart Failure Therapies (STRONG-HF) trial—a multicenter randomized, open-label, parallel-group study—evaluated if biomarkers can help optimize guideline-recommended therapies. The elected biomarkers were NT-proBNP and GDF-15, but during the study GDF-15 was removed from study design due to logistical reasons [105].

The inclusion of GDF-15 in innovative trials is demonstrative of its undisputed potential in CV risk stratification. The VICTORIA (Vericiguat Global Study in Subjects with Heart Failure with Reduced Ejection Fraction) trial conclude that, besides the reduction in the primary endpoint (CV death or HF hospitalization) with the addition of the novel drug, vericiguat, to standard of care HF treatment, GDF-15 was an independent predictor of the primary adverse outcome [106].

This premise is corroborated by the ongoing trial Dapagliflozin vErsus SacubiTrIl-valsartaN therapY in Heart Failure with reduced ejection fraction (DESTINY-HF), which explores the impact of two first-line drugs in HFrEF treatment, dapagliflozin and sacubitril/valsartan on GDF-15 values, and therefore the role of GDF-15 as a marker for therapeutic efficacy in real-world settings. Tiwari et al. consider that the potential of GDF-15 as a biomarker in HF management has not been sufficiently investigated [107].

According to the Biomarker Study Group of the HFA, controversy persists whether variations in GDF-15 represent HF progression or simply an unspecific morbid condition, forbidding its application in guided-therapy [60].

To our knowledge, there is no robust evidence that preconizes the use of GDF-15 in HF tailored treatment; so far, the conception of GDF-15 as a treatment response biomarker remains speculative. Similarly, the use of GDF-15 as a therapeutic target is merely theoretical and requires further investigation [53,60].

Based on the premise that biomarkers are products of a specific pathophysiological signaling pathway, some investigators further advanced their research by evaluating whether modulation of these pathways could serve as novel therapeutic targets to abrogate cardiac remodeling.

Assuming that myocardial fibrosis is determinant in HF genesis and prognosis the PIRfenidOne in patients with heart failUre and preserved lEfT venTricular Ejection fraction (PIROUETTE) investigators assessed the efficacy of the anti-fibrotic pirfenidone, used for the treatment of pulmonary fibrosis, in CHF patients with HFpEF and myocardial fibrosis (evaluated using cardiac magnetic resonance). Although pirfenidone reduced myocardial extracellular volume, a measure of fibrosis, after 52 weeks when compared to placebo, clinical endpoints (i.e., 6 min walk distance and diastolic function) did not improve, suggesting that fibrosis reduction per se is not sufficient to ameliorate clinical features [108].

A subsequent analyses of the PIROUETTE trial reported that the antifibrotic agent failed to influence circulating GDF-15 levels indicating that other underlying factors besides fibrosis trigger its synthesis in the HF syndrome. Nevertheless, GDF-15 was associated with several conditions, emphasizing that it could represent a proxy marker of overall frailty rather than HF severity [69].

A recent analysis of the Multi-Ethnic Study of Atherosclerosis (MESA) cohort using cardiac magnetic resonance acknowledged that GDF-15 was not related to subclinical cardiac fibrosis [109].

Further research is warranted to explore the biological effect of myocardial fibrosis reduction on HF clinical improvement and progression.

A 2024 study in animal models (i.e., mice) demonstrated that blocking GDF-15 activity halts the progression of HF and prevents cardiac cachexia, indicating that GDF-15 could be a potential therapeutic target among HF patients [110].

However in human studies, the Effects of Ponsegromab on Health-Related Quality of Life and Safety in Patients With Heart Failure (GARDEN-TIMI 74)—a phase II study that tested the inhibition of GDF-15 through a monoclonal antibody named ponsegromab, used to treat cachexia in cancer patients—was stopped prematurely since it worsened HF, suggesting that GDF-15 may play a protective role in HF, differently from cancer. Surprisingly, the composite endpoint of worsening HF event or CV death was higher in the ponsegromab arm compared to placebo, despite weight gain. The investigators justify the unexpected clinical results with the fact that there could be off-target effects resulting from GDF-15 blockage, namely the increase in systemic inflammation and the disturbance of metabolic pathways [111].

Thus, an alternative therapeutic strategy may explore the reciprocal interplay between HF and metabolic conditions, such as insulin resistance and mitochondrial dysfunction, considering the key nexus of GDF-15 in CV and metabolic pathology [24].

The divergent results across different populations regarding the correlation between GDF-15 and BMI may be justified by the paradoxical effect that GDF-15 exerts depending on individual features, acting as a protective cytokine in diabetes and obesity by refraining appetite and regulating body weight through blood glucose homeostasis, and, conversely, playing a deleterious role in elderly multimorbid patients by causing weight loss thru anorexia and reducing physical activity [112].

The GDF-15 oxymoron may have profound implications in the management of distinct diseases and culminate in contradictory and disappointing findings, as in the aforementioned clinical trials [108,111].

The cumulative scientific evidence consistently corroborates GDF-15 as a reliable independent predictor of both all-cause mortality and hospitalization in HF patients that can be used for the precocious identification of high risk patients that could benefit from a more zealous follow-up and, eventually, treatment intensification.

In the coming years, the blockade of alternative pathophysiological pathways may act as novel synergistic HF therapeutic targets, but firstly it is crucial to understand the independent role that distinct biomarkers play in this complex syndrome.

Growth differentiation factor-15 has consistently demonstrated prognostic value in HF, meaning that its circulating levels are associated with the future risk of adverse outcomes such as mortality and HF hospitalization. Numerous cohort studies and meta-analyses have shown that elevated GDF-15 concentrations independently predict poor outcomes across the spectrum of HF phenotypes, including both reduced and preserved ejection fraction. Importantly, the incorporation of GDF-15 into multi-marker strategies alongside established biomarkers such as NT-proBNP and cardiac troponins has been shown to improve risk stratification models and clinical risk scores. In contrast, the predictive value of GDF-15 (its ability to identify which patients will benefit from a specific therapy) remains uncertain. Current evidence does not demonstrate that GDF-15 levels can reliably guide therapeutic selection or predict response to guideline-directed HF therapies.

In clinical practice, if implemented, GDF-15 measurement would most plausibly occur at baseline during risk stratification or during hospitalization for AHF, potentially complemented by serial measurements during follow-up. Baseline levels may help identify patients at particularly high risk of mortality or rehospitalization, while serial assessment could provide information about disease trajectory, especially when interpreted alongside traditional biomarkers. Some studies suggest that persistent elevation or inadequate decline in GDF-15 during follow-up may reflect ongoing systemic stress, inflammation, and comorbidity burden, which are associated with worse outcomes.

However, the direct impact of GDF-15 measurement on patient management is currently limited. At present, its main potential clinical utility lies in improving risk stratification rather than guiding therapy. Identifying patients with markedly elevated GDF-15 levels could support closer monitoring, more intensive follow-up, optimization of guideline-directed therapy, or earlier referral to specialized HF programs. Nevertheless, due to its lack of tissue specificity, variability across comorbid conditions, and the absence of validated therapeutic strategies based on GDF-15 levels, routine clinical use remains premature. Large prospective studies are required to establish standardized thresholds, optimal timing of measurement, and whether GDF-15-guided strategies can meaningfully improve clinical outcomes.

## 6. Discussion

**This section will disclose the key findings, outlining the challenges and limitations associated with the incorporation of GDF-15 into routine clinical practice. Future research directions and emphasis on the potential implications of GDF-15 as a promising biomarker in the context of heart failure management will also be addressed.**

According to Castiglione et al. an ideal biomarker requires widespread testing [10], a requirement that has been fulfilled during the two decades of investigation since GDF-15 was originally divulged as a potential CV biomarker [36].

Secondly, the abovementioned authors recommend that a biomarker should have a reasonable cost, be easy to measure and present well-defined characteristics to facilitate standardization [10]. Regarding this subject, the fact that GDF-15 is essentially used for investigational purposes, rather than in daily basis clinical care, makes it less affordable than traditional biomarkers. Furthermore, the absence of well-established thresholds for risk stratification compromises this recommendation [78] and large general population studies addressing the age and sex-stratified reference ranges for GDF-15 are scant [67].

The uniformization of GDF-15 assays is also mandatory in order to facilitate its widespread use, as different quantification methods are not directly comparable [60].

Advantageous pre-analytic features enable the quantification of GDF-15 in both serum and plasma by immunoassay [24].

Another requirement of an ideal biomarker is that it should translate a fundamental pathophysiological pathway [10], based on the assumption that variations over time reproduce clinical improvement or disease progression. The ESC Biomarker Study Group of the HFA considers GDF-15 a less stable biomarker than other novel biomarkers [60] and most investigators consider that, due to its complex interplay, the physiopathological pathways of GDF-15 need further clarification [53].

Castiglione et al. also suggest that a biomarker ought to yield complementary data to those previously available [10].

The efficacy of individual biomarkers within a single time point is limited for both diagnosis and prognosis, therefore a serial multi-marker strategy combining biomarkers that represent complementary pathophysiological pathways seems to be the best strategy for HF management. There is growing evidence that GDF-15 provides valuable data when added to traditional biomarkers in multi-marker models [79,85,87].

In addition to this premise, the ideal biomarker should refine the definition of HF diagnosis, prognosis or management [10].

Kempf et al. affirmed that GDF-15 was not appropriate for diagnostic purposes since it lacked tissue specificity [37]. Table 2 portrays the advantages and disadvantages of GDF-15 in HF management.

Recent data hypothesizes that GDF-15 and other unspecific fibrosis biomarkers enable the identification of HF patients [72]; however, the Biomarker Study Group of HFA states that GDF-15 is inadequate for diagnostic purposes. This entity concludes that it is questionable if GDF-15’s fluctuations reflect HF progression or unspecific morbid condition [60].

Regarding prognosis, the addition of GDF-15 to several risk scores based on traditional biomarkers has further improved their accuracy [91,92,93,94].

Significant evidence supports GDF-15 as an independent predictor of both HF hospitalization and mortality in CHF, throughout LVEF phenotypes [79]. Also, there is robust data showing that GDF-15 is a solid predictor of these outcomes in AHF irrespective of LVEF [53,79,84,85,87,89].

Notably, GDF-15 appears to have a better prognostic performance in HFpEF individuals [73,91].

The upregulation of GDF-15 triggered by a plethora of pathological conditions could be the answer for this finding as HFpEF patients, typically, suffer from a great number of comorbidities [83].

In addition, some authors have postulated the superior prognostic capability of GDF-15 compared to NT-proBNP [89,90].

Considering HF management, guided-treatment based on GDF-15 variations during follow-up and its application as a therapeutic target are still hypothetical [41], as the effect that evidence-based HF drugs exert on circulating GDF-15 concentrations is poorly understood [113] and clinical trials targeting GDF-15 were frustrating [108,111].

Natriuretic peptides are the most extensively used biomarkers for both diagnostic and prognostic purposes, whereas their effectiveness in guiding therapy remains a matter of discussion. Even though various emerging biomarkers are under evaluation for diagnosis and risk stratification, none proved specificity for HF, and none are presently endorsed for routine clinical practice [114]. 

Presently, the Biomarker Study Group of the HFA state that the influence of therapies, age and common comorbidities on novel biomarkers, as well as the best sampling timing, need clarification [60].

In essence, despite the widespread investigation of GDF-15 in CV management, its prognostic propensity throughout the CV disease continuum needs elucidation [60,78].

Heart failure is an intricate syndrome given the multiplicity of the physiopathological mechanisms inherent to its genesis and progression, therefore it is mandatory to understand the subjacent basic science; omics arises as an undeniable link between mechanist pathways and clinical practice [60,115].

Artificial intelligence may also play an important role by integrating the sum of basic science evidence and routine data into accurate, affordable and practical risk stratification models. The future in HF management is as challenging as it is promising.

## 7. Conclusions

**The final section summarizes the *leges artis*.**

An extensive and contemporary body of evidence supports GDF-15 as an emerging and independent prognosticator of adverse outcomes throughout the wide spectrum of HF patients. However, despite two decades of intensive investigation some issues remain controversial. Large-scale prospective studies are urged to confirm the trustworthiness of GDF-15, in order to enable its general routine clinical usage.

Multivariable predictive models combining routine data and emerging biomarkers, in order to suffice HF’s complex physiopathological mechanisms and diverse etiologies, arise as a promising strategy for precocious identification of high-risk patients.

This stress cytokine appears to be a surrogate marker of disease severity, rather than reflecting any particular clinical characteristic or HF phenotype. The Biomarker Study Group of the HFA, conclude that it is questionable whether GDF-15’s fluctuations reflect unspecific morbid condition/inflammation rather than HF progression preventing tailored-treatment.

Intriguingly, drugs effective in treating cancer and pulmonary fibrosis have failed to improve HF’s outcomes suggesting that the inflammatory pathways related to HF are unique and require further clarification.

In the near future, omics approaches may help unveil the complex underlying HF physiopathological pathways; additionally, artificial intelligence may integrate the resulting data into efficient and practical risk algorithms.

**Strengths and Limitations**

We collected information from PubMed, however there are other sources of reliable data (e.g., EMBASE, Cochrane) that were not consulted which could implicate missing some important papers. Nevertheless, we believe that the fundamental studies were addressed, since a comprehensive selection of state-of-the-art articles was conducted, supplemented by the original GDF-15 studies, and through a manual analysis of bibliographic references for related studies. Moreover, studies from different countries and continents were included, enabling a transversal comprehension of the subject.

Several systematic reviews and meta-analyses were considered, permitting us to synthetize several studies and to reconcile some controversial topics in the literature.

## Figures and Tables

**Table 1:** Trials that addressed the prognostic utility of GDF-15.

Study	Setting	Population (*n*)	Primary Endpoint	Follow-Up
Kempf et al. [37]	CHF	455 patients. Predominantly HFrEF. Median LVEF: 32% (IQR 25–39%). HFpEF and HFmrEF: not reported	All-cause mortality	Median of 40 months (IQR 14–78 months)
Ceelen et al. [53]	CHF	2279 patients in the BIOSTAT-CHF index cohort. 1705 patients in the validation cohort. Not reported as HFrEF/HFmrEF/HFpEF categories. Index cohort: LVEF 30.0% [25.0–35.0] (lowest GDF-15 quartile) vs. 30.0% [21.0–37.0] (highest quartile). Validation cohort: LVEF 39.5% [35.0–48.0] (lowest quartile) vs. 40.0% [35.0–50.0] (highest quartile)	2-year all-cause mortality and 2-year HF hospitalization	2 years
Teramoto et al. [47]	CHF	942 hypertensive patients with compensated HF (93% NYHA II). Mixed LVEF population: HFrEF (LVEF ≤ 40%): 17.2%; HFmrEF (LVEF 41–49%): 18.6%; HFpEF (LVEF ≥ 50%): 64.2%. Median LVEF (overall): 56% (IQR 45–66)	Composite endpoint of HF hospitalization or all-cause mortality	Mean of 8.6 years
Anand et al. (Val-HeFT) [44]	CHF	1734 patients (*Val-HeFT*). Predominantly HFrEF (LVEF ≤ 40%). Mean LVEF: 26 ± 7%. HFpEF and HFmrEF: not included	All-cause mortality/First morbid event (first morbid event defined as death, sudden death with resuscitation, HF hospitalization, or IV inotropic/vasodilator therapy without hospitalization)	Median of 23 months
Sharma et al. (HF-ACTION) [80]	CHF	910 patients (HF-ACTION substudy). HFrEF only (LVEF ≤ 35%). Median LVEF: 24.7% (IQR 20.0–30.1). HFpEF and HFmrEF: not included	All-cause death or all-cause hospitalization/CV death or HF hospitalization	Median of 30 months
Bouabdallaoui et al. (PARADIGM-HF) [64]	CHF	1935 patients (PARADIGM-HF). HFrEF only (LVEF ≤ 40% amended to ≤35%). Mean LVEF: 30 ± 6% HFpEF/HFmrEF: not included	Combined endpoint of cardiovascular death or hospitalization for HF	Mean of 27 months
McDowell et al. (PARADIGM-HF) [81]	CHF	1559 patients (PARADIGM-HF). HFrEF (LVEF ≤ 40%). Mean LVEF 30.7 ± 6%). HFpEF and HFmrEF: not included	CV death or HF hospitalization	Mean of 30.7 months
Ferreira et al. (EMPEROR program) [82]	CHF	1124 patients (EMPEROR program). Mixed population: HFrEF (EMPEROR-Reduced) and HFpEF (EMPEROR-Preserved). Overall LVEF (entire cohort): 40 ± 15%. Patients with LVEF < 50%: ≈69%. HFmrEF not analyzed as a separate category	Cardiovascular death or HF hospitalization	EMPEROR-Reduced Mean of 16 months EMPEROR-Preserved Mean of 26 months
Lyu et al. [91]	CHF	823 patients. Mixed LVEF population: HFrEF 230 patients (27.9%); HFmrEF: 271 patients (32.9%) and HFpEF: 322 patients (39.1%). Median LVEF (overall): 46% (IQR 40–55)	All-cause mortality and HF rehospitalization	Median of 9.4 years
Miftode et al. [73]	AHF	173 patients (120 hospitalized with AHF and 53 CHF controls). Mixed EF population: includes HFrEF and HFpEF. Mean LVEF (AHF group): 33.8 ± 13.9%. Specific proportions of HFrEF, HFmrEF and HFpEF not reported	30-day all-cause mortality (including in-hospital all-cause mortality)	During hospitalization and 30 days after admission
Cotter et al. (RELAX-AHF) [84]	AHF	1161 patients (RELAX-AHF). Mixed AHF population: includes both HFpEF and HFrEF. Mean LVEF: 38.5 ± 14.5%. Specific proportions of HFrEF, HFmrEF, and HFpEF: not reported	Composite endpoint of HF or renal failure readmission at 60 days/CV death or CV death at 180 days	60 days for HF/renal failure readmission and 180 days for cardiovascular mortality
Gürgöze et al. [87]	AHF	475 patients with HF included in the final analysis. Mixed AHF population, includes both HFpEF and HFrEF. Median LVEF: 30% (IQR 21–41). HFrEF: 83% (ESC 2012 definition)/69% (ESC 2016 definition). HFpEF and HFmrEF: included but not analyzed separately	Composite of all-cause mortality and HF hospitalization	Median follow-up of 325 days (IQR 85–401); maximum follow-up up to 400 days
Kosum et al. [88]	AHF	84 patients. Mixed AHF population: HFpEF (LVEF ≥ 50%): 52.3%; HFmrEF (LVEF 40–49%): 7.7% and HFrEF (LVEF < 40%): 23.1%	30-day HF rehospitalization or 30-day all-cause mortality	Median of 213 days
Płonka et al. [79]	AHF	104 patients. Mixed AHF population. Majority HFrEF (LVEF < 40%). HFpEF and HFmrEF: included but not analyzed as separate categories. Median LVEF: 30% (IQR 20–38)	All-cause mortality or HF rehospitalization at 1-year follow-up	1 year
Yin et al. [89]	AHF	380 patients. HFpEF only (LVEF ≥ 50% at admission). Median LVEF: 59% (IQR 53–65). HFrEF and HFmrEF: not included	1-year all-cause mortality and 1-year HF readmission	1 year
Cortes et al. [90]	AHF	104 patients. HFrEF only (LVEF ≤ 40%). Median LVEF: 20% (IQR 15–20). HFpEF and HFmrEF: not included	All-cause mortality	Median of 23.5 months
Hongisto et al. (CardShock) [92]	AHF (cardiogenic shock)	177 patients with cardiogenic shock. Mixed cardiogenic shock population: includes both HFpEF and HFrEF. Mean LVEF: 33 ± 14%. Proportions of HFrEF, HFmrEF and HFpEF: not reported	90-day mortality	90 days after detection of cardiogenic shock
Sakamoto et al. (PURSUIT-HFpEF) [93]	AHF	1100 patients (PURSUIT-HFpEF). HFpEF only (LVEF ≥ 50%). Median LVEF at discharge: ≈64%. HFrEF and HFmrEF: not included	Composite of all-cause death and HF hospitalization	Median of 733.5 days (IQR 397.8–1108.0 days)

IQR—interquartile range; IV—intravenous.

**Table 2:** Advantages and disadvantages of GDF-15 in HF management.

Advantages	Disadvantages
Widespread testing	Basically used for investigational purposes
Advantageous pre-analytic features	Physiopathological pathways needs further clarification
Incremental value of routine practice	Less stable biomarker than other novel biomarkers
	Lacks tissue specificity
	Absence of well-established thresholds
	Best sampling timing needs clarification

## Data Availability

The analyzed data, compiled from existing literature, is publicly accessible through the respective digital object identifier. Data sharing is not applicable to this article as no new datasets were generated or analyzed.

## Abbreviations

The following abbreviations are used in this manuscript:

ACC	American College of Cardiology
AHA	American Heart Association
AHF	Acute Heart Failure
BMI	Body Mass Index
BNP	B-type natriuretic peptide
CHF	Chronic Heart Failure
CV	Cardiovascular
eNOS	Endothelial Nitric Oxide Synthase
ERK1/2	Extracellular Signal-Regulated Kinases 1/2
ESC	European Society of Cardiology
FGF-23	Fibroblast Growth Factor 23
Gal-3	Galectin-3
GDF-15	Growth differentiation factor-15
GDFN	Glial Cell Line-Derived Neurotrophic Factor
GFRAL	GDNF family receptor alpha-like
HF	Heart Failure
HFA	Heart Failure Association
HFmrEF	Heart Failure with Mildly Reduced Ejection Fraction
HFpEF	Heart Failure with Preserved Ejection Fraction
HF	Heart Failure with Reduced Ejection Fraction
hs-CRP	High-sensitivity C-Reactive Protein
hs-cTnT	High-sensitivity Cardiac Troponin T
IGF-1	Insulin-like Growth Factor
IL-1	Interleukin-1
IL-6	Interleukin-6
ISR	Integrated Stress Response
IQR	Interquartile Range
IV	Intravenous
LILRB4	Leucocyte Immunoglobulin-Like Receptor Subfamily B Member 4
LV	Left Ventricle
LVEF	Left Ventricular Ejection Fraction
MeSH	Medical Subject Headings
MIC-1	Macrophage Inhibitory Cytokine
MMP	Matrix Metalloproteinases
NF-κB	Nuclear Factor Kappa-Light-Chain-Enhancer of Activated B Cells
NT-proBNP	N-Terminal pro-B-type Natriuretic Peptide
NYHA	New York Heart Association
PI3K/Akt	Phosphoinositide 3-Kinase/Protein Kinase B
PLAB	Placental Bone Morphogenetic Protein
pTGFB	Placental Transforming Growth Factor-Beta
RAAS	Renin–Angiotensin–Aldosterone System
ROC	Receiver Operating Characteristic
SMAD	Suppressor of Mothers Against Decapentaplegic Homolog
SNS	Sympathetic Nervous System
SOD	Superoxide Dismutase
sST2	Soluble Isoform of Suppression of Tumorigenicity 2
TFF3	Trefoil Factor 3
TGF-β	transforming growth factor-beta
TIM	Tissue Inhibitors of Metalloproteinases
TNF-α	Tumor Necrosis Factor-Alpha
TNFRSF11A	Tumor Necrosis Factor Receptor Superfamily Member 11a
TRAIL-R2	Tumor Necrosis Factor-Related Apoptosis-Inducing Ligand Receptor 2
WISP-1	WNT1-Inducible Signaling Pathway Protein 1
